# The prevalence of non-GII.4 norovirus genotypes in acute gastroenteritis outbreaks in Jinan, China

**DOI:** 10.1371/journal.pone.0209245

**Published:** 2018-12-28

**Authors:** Lanzheng Liu, Hengyun Guan, Ying Zhang, Chunrong Wang, Guoliang Yang, Shiman Ruan, Huailong Zhao, Xiuyun Han

**Affiliations:** 1 Viral Disease Inspection Laboratory, Jinan Center for Disease Control and Prevention, Jinan, China; 2 Sydney School of Public Health/China Studies Center, The University of Sydney, Sydney, NSW, Australia; 3 Shandong University Climate Change and Health Center, Shandong University, Shandong, China; Universita degli Studi di Parma, ITALY

## Abstract

Noroviruses (NoVs) are the leading cause of acute viral gastroenteritis outbreaks. From June 2015 to March 2017, fifteen outbreaks of acute gastroenteritis (AGE) were reported to the Jinan Center for Disease Control and Prevention in China. To identify the circulating NoV genotypes associated with outbreaks in Jinan, China, 414 specimens from the 15 outbreaks were collected and analyzed for the causative viruses, and phylogenetic analysis was performed on the NoV-positive strains. The NoV detection rate was 57.5% (238/414), and a total of 14 outbreaks were caused by NoVs (eight by infection with genogroup II (GII), five by mixed infection with GI and GII, and one by mixed infection with GII and rotavirus (RoV)-A). A total of 75 NoV sequences were obtained from 13 NoV-positive outbreaks and classified into seven genotypes (38 GII.17, 13 GII.2, 4 GII.3, 4 GII.1, 10 GI.6, 5 GI.5 and 1 GI.3), while GII.4 was not identified. The most prevalent genotype changed yearly during the 2015–2017 period. Phylogenetic analysis demonstrated that these NoV genotypes had high homology with the strains circulating worldwide, especially strains from Asian countries and cities. Our study illustrated that multiple non-GII.4 NoV genotypes were prevalent in outbreaks of AGE in Jinan, China. Year-round surveillance of multiple NoV genotypes could help health authorities reduce the impact of NoV outbreaks on public health.

## Introduction

Noroviruses (NoVs) are the principal causative pathogen of nonbacterial acute gastroenteritis (AGE) among five viral agents (NoV, sapovirus (SaV), rotavirus (RoV), astrovirus (Ast) and human adenovirus (ADV)) [1]. Globally, NoVs are estimated to be associated with 18% of all cases of AGE [2]. NoV outbreaks have been reported in congregate settings, such as cruise ships, childcare centers, and long-term care facilities [3–5]. The common symptoms are nonbloody diarrhea and/or vomiting accompanied by low-grade fever, abdominal pain, and nausea. Dehydration is the most frequent and dangerous complication [6]. Outbreaks caused by NoV result in a total of $4.2 billion in direct health care system costs and $60.3 billion in annual societal costs worldwide [7]. NoV transmission mainly occurs through foodborne and human-to-human transmission via the fecal-oral and vomit-oral routes [8]. A low infectious dose facilitates effective NoV transmission. The estimated 50% infectious dose (ID_50_) ranges from 18 to 2800 genomic equivalents [9–10].

NoVs are members of the *Caliciviridae* family and possess a 7.5–7.7 kb genome. NoVs are nonenveloped, single-stranded positive-sense RNA viruses. To date, NoVs have been genetically classified into seven genogroups (GI to GVII) and thirty-nine genotypes, including 9 genotypes in GI, 22 in GII, 2 in GIII, 2 in GIV, 1 in GV, 2 in GVI, and 1 in the tentative new GVII genogroup [11]. GII.4 has been the predominant genotype associated with gastroenteritis outbreaks and sporadic cases worldwide in the past two decades [12]. However, since 2014, novel non-GII.4 NoV genotypes continue to be reported in outbreaks. In the winter of 2014, GII.17 became the newest circulating genotype among gastroenteritis outbreaks originating in Asia [13–15]. In addition, a new recombinant NoV, GII.P16-GII.2, was frequently identified in Germany in November 2016 [16]. Whether the dominant GII.4 strains will be replaced by non-GII.4 NoV genotypes worldwide remains to be seen [17].

Epidemics of NoV outbreaks have increased substantially in China since the winter of 2014 [18]. Qin and colleagues reported that NoV outbreaks showed an increasing trend from 2006–2016 in China. Only 0.8% (1/132) of the identified NoV outbreaks were reported in 2006, whereas 46.2% (61/132) occurred in 2015, and 11.4% (15/132) occurred in the first half of 2016 [19]. The Guidelines on Outbreak Investigation, Prevention and Control of Norovirus Infection (2015) were released in 2015 to standardize the surveillance of NoV outbreaks [20]. Jinan is the capital city of Shandong Province in Eastern China and has a total population of seven million. A series of AGE outbreaks have been reported to the Jinan Center for Disease Control and Prevention (CDC) since June 2015. In the present study, we aimed to determine the circulating NoV genotypes associated with AGE outbreaks in Jinan, China. We further explored the epidemiological characteristics of the outbreaks that occurred from June 2015 to March 2017. This study should help health authorities and policy makers create effective measures to address NoV outbreaks.

## Materials and methods

### Ethics statement

This study was approved by the Ethics Committee at the Jinan CDC in China. Verbal informed consent was obtained from all study participants or, in the case of minors, from the parents. Information on individual patients and organizations was anonymized during data analysis.

### Epidemiological surveillance and specimen collection

According to the Guidelines on Outbreak Investigation, Prevention and Control of Norovirus Infection (2015), an AGE outbreak was defined as the occurrence of at least 20 cases, including at least two laboratory-confirmed cases, within one week from a common exposure [20]. According to the guidelines, outbreaks are required to be reported to the National Emergent Public Health Event Information Management System. A field investigation is then performed, and data are collected via a questionnaire survey. The first reported individual in an outbreak is regarded as the index case. For this study, the human specimens (stool, anal swab, vomit or throat swab) and/or environmental specimens (food, water or surface swab) were collected and delivered to the Jinan CDC using refrigerated transport at 4°C. The human specimens were collected from clinically compatible cases within 5 days of onset. Viral detection was performed on the specimens in the Viral Disease Detection Laboratory at the Jinan CDC. The virus-negative specimens were sent to the Department of Microbiology at the Jinan CDC for bacterial testing.

### RNA/DNA extraction

The stool, anal swab, vomit, and food samples were processed in a 10% suspension of phosphate-buffered saline (supplemented with Mg^2+^ and Ca^2+^). The throat and surface swabs were stored in Hank’s balanced salt solution. Contaminated water was concentrated using a centrifugal filter (Merck Millipore, Ltd., Ireland). All samples were centrifuged at 8000 rpm for 10 min (Beckman Coulter, Inc., USA). RNA/DNA was extracted from 200 μl supernatant using the MagNA Pure LC Total Nucleic Acid Isolation Kit (Roche, Mannheim, Germany) according to the manufacturer’s instructions and suspended in 50 μl elution buffer. RNA/DNA was amplified immediately or stored at −80°C°C. The protocol is provided as DOI: dx.doi.org/10.17504/protocols.io.srjed4n.

### Detection of causative viruses

To identify the causative agents of these outbreaks, detection of five viruses closely associated with AGE was performed according to the Viral Diarrhea Surveillance Scheme in Shandong Province. NoV, RoV, SaV and Ast were detected by real-time RT-PCR; ADV was detected by real-time PCR (Bioperfectus Technologies Co., Jiangsu, China) on a Stratagene Mx3005P system (USA) [20]. The amplification conditions were according to the manufacturer’s instructions. The virus-negative samples were sent to the Department of Microbiology at the Jinan CDC for bacterial testing.

### NoV genotyping

NoV-positive specimens were further genotyped using specific primers targeting a partial sequence (region C) of the gene encoding the major capsid protein (VP1) in the NoV genome. As Yan et al. described, instead of the complete VP1 gene, a relatively small region of ORF2 (region C) is generally used to genotype strains [21]. The primer sequences were as follows: G1-SKF (CTG CCC GAA TTY GTA AAT GA) and G1-SKR (CCA ACC CAR CCA TTR TAC A) for GI [22] and COG2F (CAR GAR BCN ATG TTY AGR TGG ATG AG) and G2SKR (CCR CCN GCA TRH CCR TTR TAC AT) for GII [22–23]. RT-PCR was performed using a SuperScript III One-Step RT-PCR System kit (Invitrogen, USA) in a 25 μl reaction volume. A negative control sample containing water and a positive control sample containing GI and GII NoV RNA provided by Bioperfectus Technologies Co. were included in each run. The amplification conditions were as follows: 42°C for 30 min; 95°C for 15 min; 40 cycles at 95°C for 30 s, 50°C for 30 s, and 72°C for 30 s; and a final extension at 72°C for 10 min. The expected amplicon size was 330 bp for GI and 387 bp for GII. The amplification products were sequenced using an ABI 3730 XL DNA analyzer (Applied Biosystems, Inc., Foster City, CA) at Beijing Genomics Institute (BGI) in Beijing, China. Genotypes were determined using the Norovirus Typing Tool (http://www.rivm.nl/mpf/typingtool/norovirus/) [24]. The protocol is provided as DOI: dx.doi.org/10.17504/protocols.io.q3sdyne.

### Phylogenetic analysis

The NoV sequences obtained from the outbreaks were analyzed based on the sequence of the VP1 region by the Molecular Evolutionary Genetic Analysis version 7.0 (MEGA7) program [25]. The neighbor-joining (N-J) method was used for phylogenetic reconstruction, and the Kimura 2-parameter model was used to compute evolutionary distances. The bootstrap analysis was performed with 1000 replications [26]. The reference strains for GI and GII were obtained from the GenBank database to demonstrate the relationships between NoV genotypes from the outbreaks and globally circulating strains.

### Statistical analysis

Statistical analyses were performed using Statistical Package for Social Sciences (SPSS v17.0) software (SPSS Inc., Chicago, IL, USA). The chi-square test was used to measure differences in the NoV infection rate among groups. Statistical significance was set at *P*<0.05.

### Accession numbers of the NoV strains

The original NoV sequences from this study were deposited in the GenBank database under the following accession numbers: KU724080-KU724082, MG871408-MG871413, MH106750-MH106779, and MH106986-MH107008.

## Results

### Epidemiological features

A total of 15 AGE outbreaks were reported to the Jinan CDC from June 2015 to March 2017. Of the 15 outbreaks, 4 (26.7%) occurred in kindergartens, 2 (13.3%) in high schools, 6 (40.0%) in colleges, 2 (13.3%) in residential districts and 1 (6.7%) at a construction site (Table 1). According to the field investigation reports, a total of 1194 people were affected in these 15 outbreaks. The index cases were enrolled into the field investigations of these outbreaks.

**Table 1:** Epidemiological features of acute gastroenteritis outbreaks in Jinan, 2015–2017.

Outbreak	Site	Date of index case onset	Duration of outbreak (d)	Number of affected people	Age range (median age) (y)	Human specimens^a^		Environmental specimens^b^	
						Total number	Number positive	Total number	Number positive
I	A college	10 June 2015	12	524	18–50 (20)	177	128	43	0
II	B college	18 June 2015	2	21	20 (20)	3	3	NS	NS
III	C college	17 June 2015	2	22	17–18 (17.5)	2	2	NS	NS
IV	D construction site	13 June 2015	5	22	23–64 (37)	13	10	15	1
V	B college	20 October 2015	5	88	18–21 (19)	8	6	NS	NS
VI	E kindergarten	20 December 2015	5	21	5–6 (6)	15	12	NS	NS
VII	F college	3 March 2016	7	253	18–23 (20)	24	18	8	2
VIII	A college	21, March 2016	5	53	18–21 (20)	7	7	NS	NS
IX	G high school	24 April 2016	5	23	17 (17)	6	5	NS	NS
X	H residential district	12 May 2016	14	24	6–56 (53)	5	2	12	8
XI	I high school	17 October 2016	5	48	16–18 (17)	26	0	NS	NS
XII	J residential district	7 December 2016	2	21	7–11 (10)	10	3	NS	NS
XIII	K kindergarten	13 February 2017	8	20	3–5 (4)	13	11	7	0
XIV	L kindergarten	17 February, 2017	5	31	3–5 (4)	12	12	NS	NS
XV	M kindergarten	2 March, 2017	8	23	3 (3)	8	8	NS	NS

NS: no samples.

^a^ Human specimens include stool samples, anal swabs, throat swabs, and/or vomit samples.

^b^ Environmental specimens include food samples, water samples and/or surface swabs.

Viral detection was performed on 414 specimens. In total, 329 human specimens were collected, including 95 stool samples, 213 anal swabs, 13 throat swabs and 8 vomit samples. A total of 85 environmental specimens were obtained from 47 food samples, 26 contaminated water samples and 12 surface swabs. All 414 specimens were tested for NoV, RoV, SaV, Ast, and ADV.

Five of the outbreaks (outbreaks I-III and VII-VIII), had one common characteristic: a farmer’s market was located near the affected colleges, at which the students often ate. During the field investigation, the affected individuals were surveyed about the food items consumed using a questionnaire; the survey indicated that most of the infected cases had a history of eating at the farmer’s market. We believe that the colleges were constantly at risk of foodborne infection due to the absence of regulations on farmer’s markets. The infected cases were not isolated at the college in a timely manner, and transmission occurred via the human-to-human route. The lack of effective supervision of colleges made it more difficult to control the outbreaks in a timely manner.

Among five of the other outbreaks, four occurred in kindergartens (outbreaks VI and XIII-XV), and one (outbreak V) occurred at a college. The field investigation showed that the affected individuals came to the kindergartens or college and without any rest. Therefore they came into close contact with others at the kindergartens or college. Using laboratory testing, NoVs were detected in samples from these affected cases. NoVs were easily transmitted via close contact among the children/college students.

Outbreak IV occurred at construction site D. The affected individuals ate lunch and supper in the construction site cafeteria. A field investigation of employees, food handlers and the sanitation conditions of the cafeteria was conducted. Because the leftovers had been cleaned up, the food samples were not collected. NoVs were identified in the ground sewage from the cafeteria, but no NoVs were detected in barreled drinking water. Outbreak X occurred in residential district H, where household tap water was supplied from a well. The well was located at a lower elevation than the residential district, and two possible contamination sources rendered the well water at a high risk of contamination. A lavatory was located approximately 30–40 meters east of the well and a vegetable garden was beside the top of the well. NoVs were detected in both the well water and the household tap water. Thus, collecting related water samples is important for analyzing the risk factors in outbreaks.

### Virological screening

**Fig 1:**
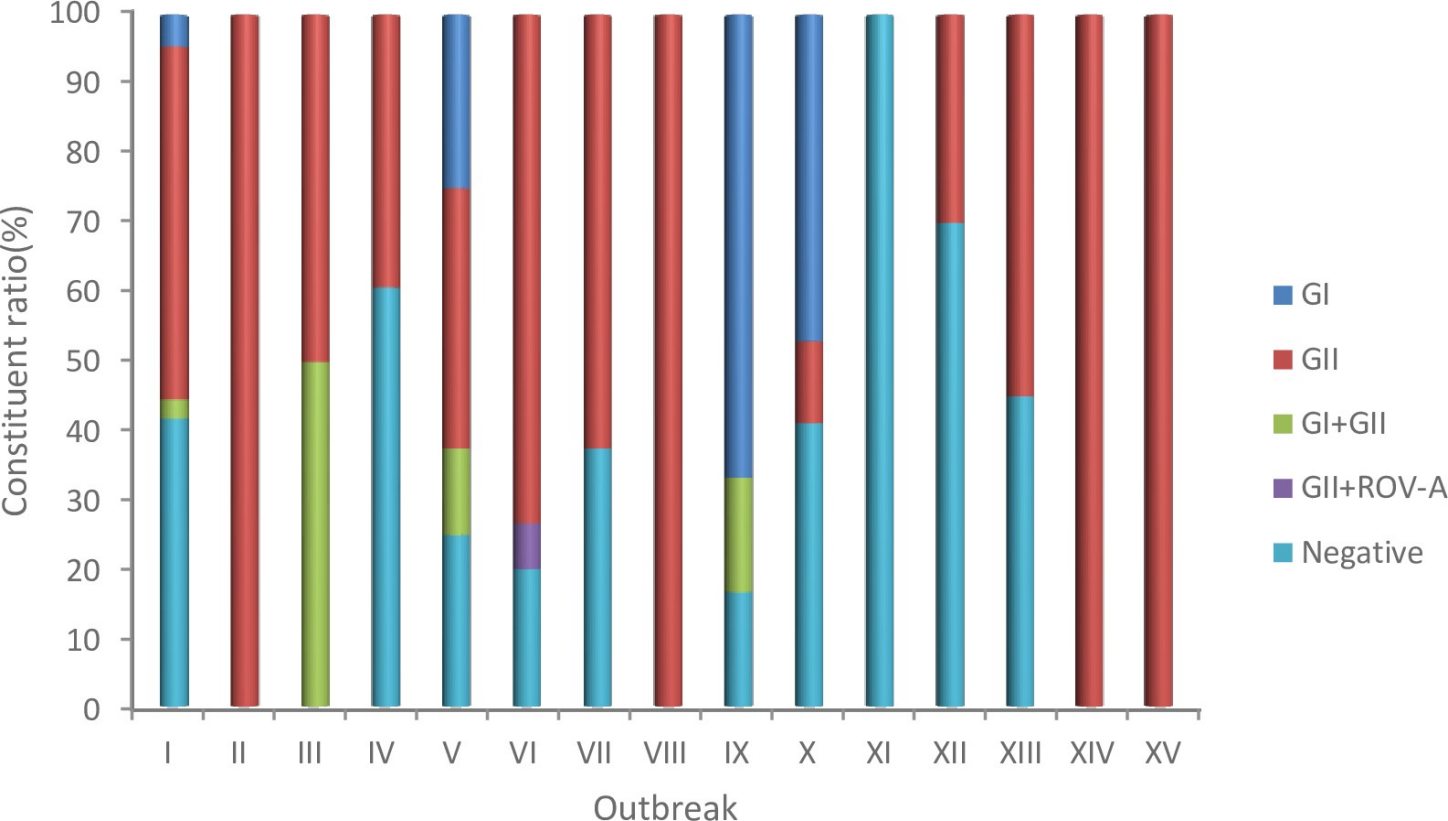
Virological screening of 15 outbreaks of acute gastroenteritis in Jinan, China, 2015–2017. “Negative” indicates that the specimens from the outbreaks were negative for NoV, RoV, SaV, Ast, and ADV.

A total of 238 of the 414 (57.5%) specimens collected from these outbreaks were positive for NoV by real-time RT-PCR. GII was the main genogroup (204/238, 85.7%) identified, followed by GI (24/238, 10.1%), dual infection with GI+GII (9/238, 3.8%) and dual infection with GII+RoV-A (1/238, 0.4%). Of the 15 outbreaks, eight were caused by GII, five were caused by mixed infections with GI and GII and one was caused by mixed infections with GII and RoV-A. Outbreak XI was later confirmed as a bacterial infection (Fig 1). All 414 specimens were negative for SaV, Ast and ADV.

Of the six types of specimens collected, fecal specimens (including stool specimens and anal swabs) had the highest detection rate (72.4%, 223/308), followed by vomit (50.0%, 4/8), contaminated water (34.6%, 9/26) and food (4.3%, 2/47). All throat swabs and surface swabs were negative.

Among the 329 human cases, the average age was 18.6 years (SD±9.1; range 3–64). Of the 329 cases, 227 (69.9%) were NoV-positive. In this study, the 329 cases were divided into five age groups. The detection rates were highest in children aged 3–6 years. No statistically significant differences were found between sexes (*P*>0.05). The outbreaks occurred year-round in Jinan, but the peak infection rate increased from the winter of one year to the summer of the following year (Table 2).

**Table 2:** Norovirus detection rates in human specimens by age, sex and season, June 2015-March 2017.

Characteristic	Group	Number of positive cases/tested cases (%)^a^	X^2^	*P* value
Age (years)	3–6	43/49 (87.8)	17.270	0.002
	7–15	3/10 (30.0)		
	16–25	164/248 (66.1)		
	26–50	15/18 (83.3)		
	≥51	2/4 (50.0)		
Sex	Male	124/181 (68.5)	0.045	0.832
	Female	103/148 (69.6)		
Season^b^	Spring	40/50 (80.0)	47.601	<0.0001
	Summer	143/195 (73.3)		
	Autumn	6/34 (17.6)		
	Winter	38/50 (76.0)		

^a^ The number of positive cases includes one with dual infection with GII+RoV-A.

^b^ Spring: March-May, Summer: June-August, Autumn: September-November, Winter: December-February

### Norovirus genotyping

To determine the NoV genotypes responsible for the outbreaks, nucleotide sequencing of region C was conducted on the NoV-positive specimens. A total of 75 strains were obtained from 13 NoV-positive outbreaks. These 75 NoV strains belonged to seven genotypes, namely, GII.17 (38, 50.7%), GII.2 (13, 17.3%), GI.6 (10, 13.3%), GI.5 (5, 6.7%), GII.3 (4, 5.3%), GII.1 (4, 5.3%) and GI.3 (1, 1.3%). Of these strains, 93.3% (70/75) were identified from faeces, 5.3% (4/75) from contaminated water and 1.3% (1/75) from vomit. No strains were identified from throat swabs, food samples, or surface swabs.

A single outbreak can be caused by multiple NoVs genotypes (Table 3). GII.17 and GI.6 were responsible for outbreak I; GII.17 and GI.3 for outbreak III; and GII.3 and GI.6 for outbreak V. For outbreak X, GI.5 was detected in both human specimens and water specimens (well water and household tap water). The well water was the standard water supply for residential district H, and contamination of this water was believed to have caused outbreak X.

**Table 3:** Norovirus genotypes identified in outbreaks of acute gastroenteritis in Jinan, 2015–2017.

Origin of specimen	Outbreak														
	I	II	III	IV	V	VI	VII	VIII	IX	X	XI	XII	XIII	XIV	XV
Human	GII.17, GI.6	GII.17	GII.17, GI.3	GII.17	GII.3, GI.6	GII.17	GII.3	GII.17	GI.6	GI.5	ND	ND	GII.2	GII.2	GII.1
Surface swab	NS	NS	NS	ND	NS	NS	NS	NS	NS	NS	NS	NS	ND	NS	NS
Water	ND	NS	NS	ND	NS	NS	NS	NS	NS	GI.5	NS	NS	NS	NS	NS
Food	ND	NS	NS	NS	NS	NS	ND	NS	NS	NS	NS	NS	ND	NS	NS

ND: not detected, NS: no samples

NoV outbreaks at a given college were associated with different NoV genotypes. Two outbreaks (outbreaks I and VIII) occurred at college A. Outbreak I, in 2015, was caused by a mixed infection with GII.17 and GI.6; however, outbreak VIII, in 2016, was a single infection with GII.17 (Table 3). Outbreaks II and V occurred at college B in 2015. GII.17 was responsible for outbreak II, and GII.3 and GI.6 were the causative agents of outbreak V.

**Fig 2:**
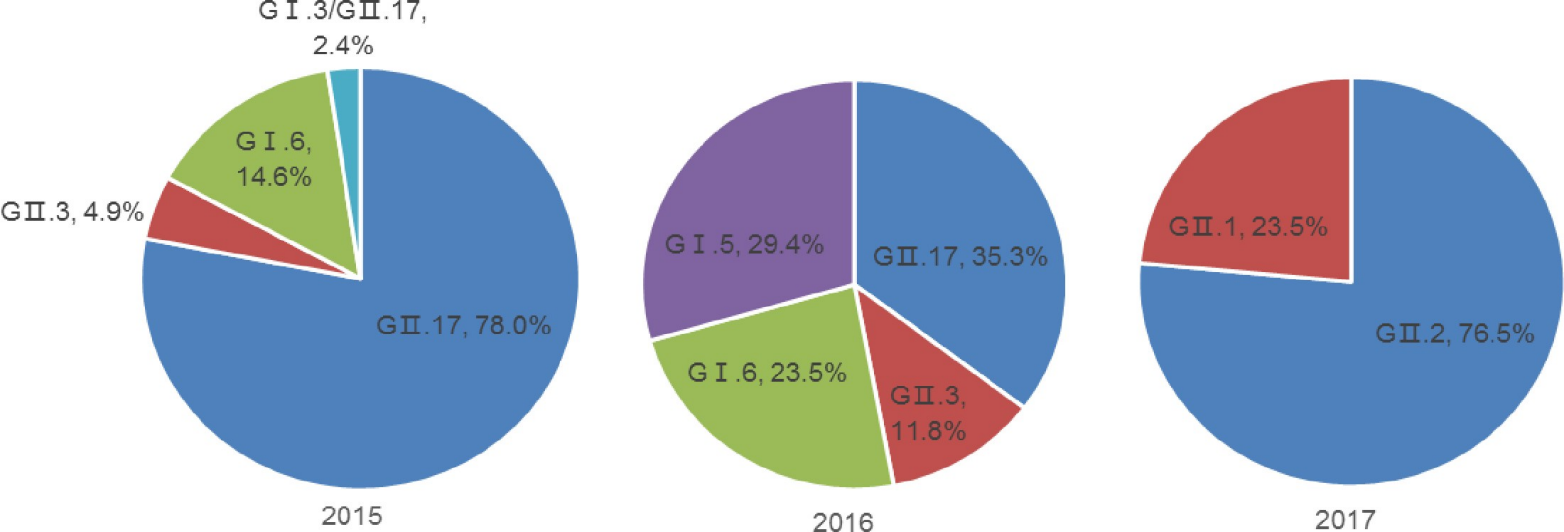
Circulating norovirus genotypes in acute gastroenteritis outbreaks in Jinan, China, June 2015-March 2017.

The circulating NoV genotypes differed from June 2015 to March 2017. GII.17 was the major genotype (n = 32; 78.1%) in 2015, four NoV genotypes (GII.17, GII.3, GI.5, and GI.6) coexisted in 2016, and GII.2 and GII.1 appeared in the first quarter of 2017 in Jinan (Fig 2).

### Phylogenetic analysis

**Fig 3:**
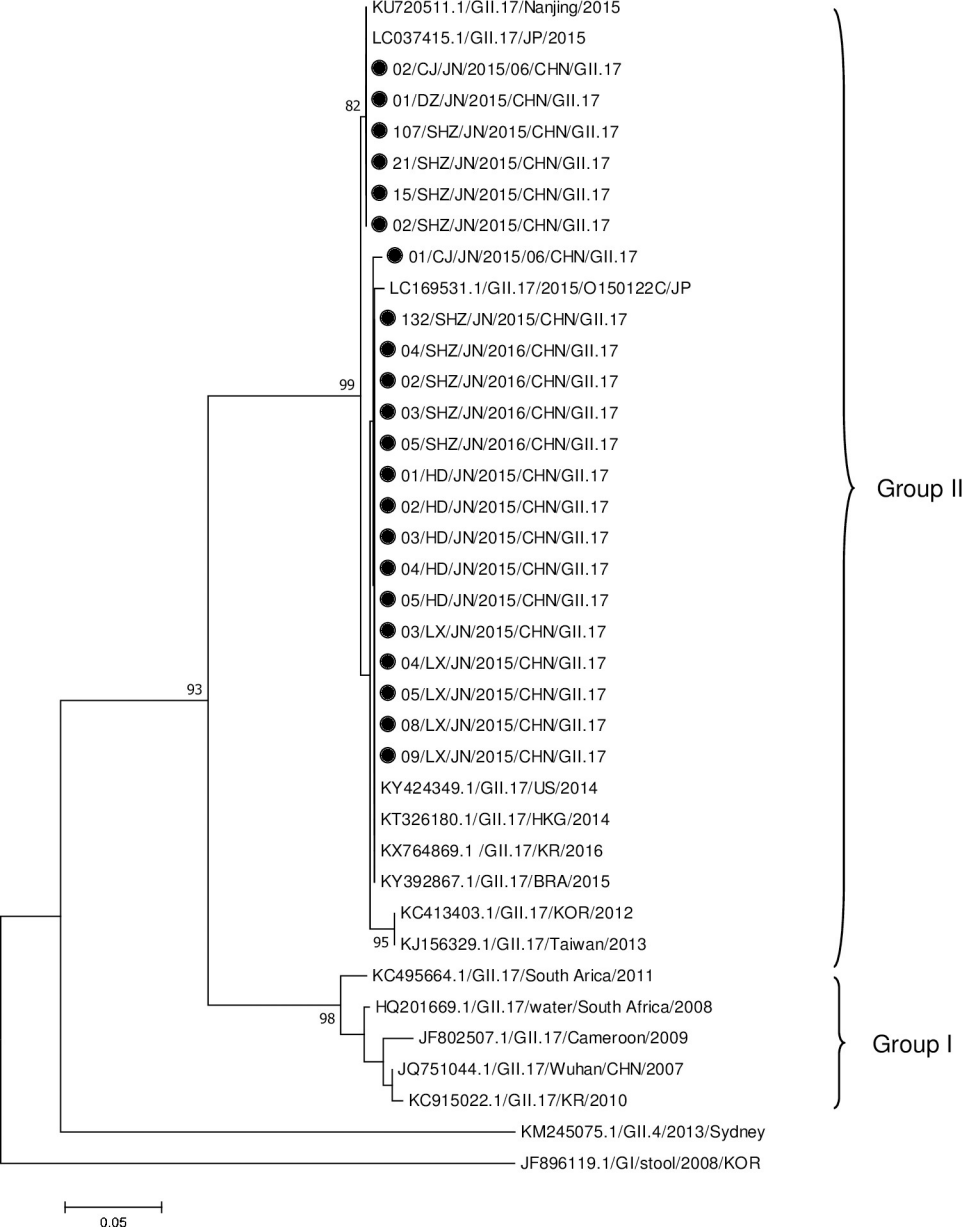
Phylogenetic analysis of the GII.17 norovirus strains isolated from outbreaks of acute gastroenteritis based on the partial sequence of the capsid gene encoding the VP1 protein. The phylogenetic tree was constructed using the neighbor-joining (N-J) method with the Kimura 2-parameter model and 1000 bootstrap replications. Bootstrap values are shown in the phylogenetic tree; values less than 80% are not represented. The bar at the bottom of the figure is proportional to the genetic distances. The black dot represents the GII.17 strains obtained from the outbreaks in Jinan in 2015–2016.

NoV GII.17 was the predominant genotype appearing in outbreaks during 2015–2016 in Jinan, China. The representative GII.17 reference strain sequences, which were identified during 2007–2016, were obtained from the GenBank database. Phylogenetic analysis of the reference strains demonstrated that the GII.17 strains were divided into two groups (Fig 3): the GII.17 strains isolated in 2007–2011 belonged to Group I, and those isolated in 2012–2016 belonged to Group II. All GII.17 strains responsible for the outbreaks in Jinan belonged to Group II. Furthermore, the GII.17 strains isolated from the Jinan outbreaks shared a higher nucleotide homology with the reference strains isolated in Nanjing in 2015 (KU720511) and Korea in 2016 (KX764869) than with the strain isolated in Korea in 2012 (KC413403).

**Fig 4:**
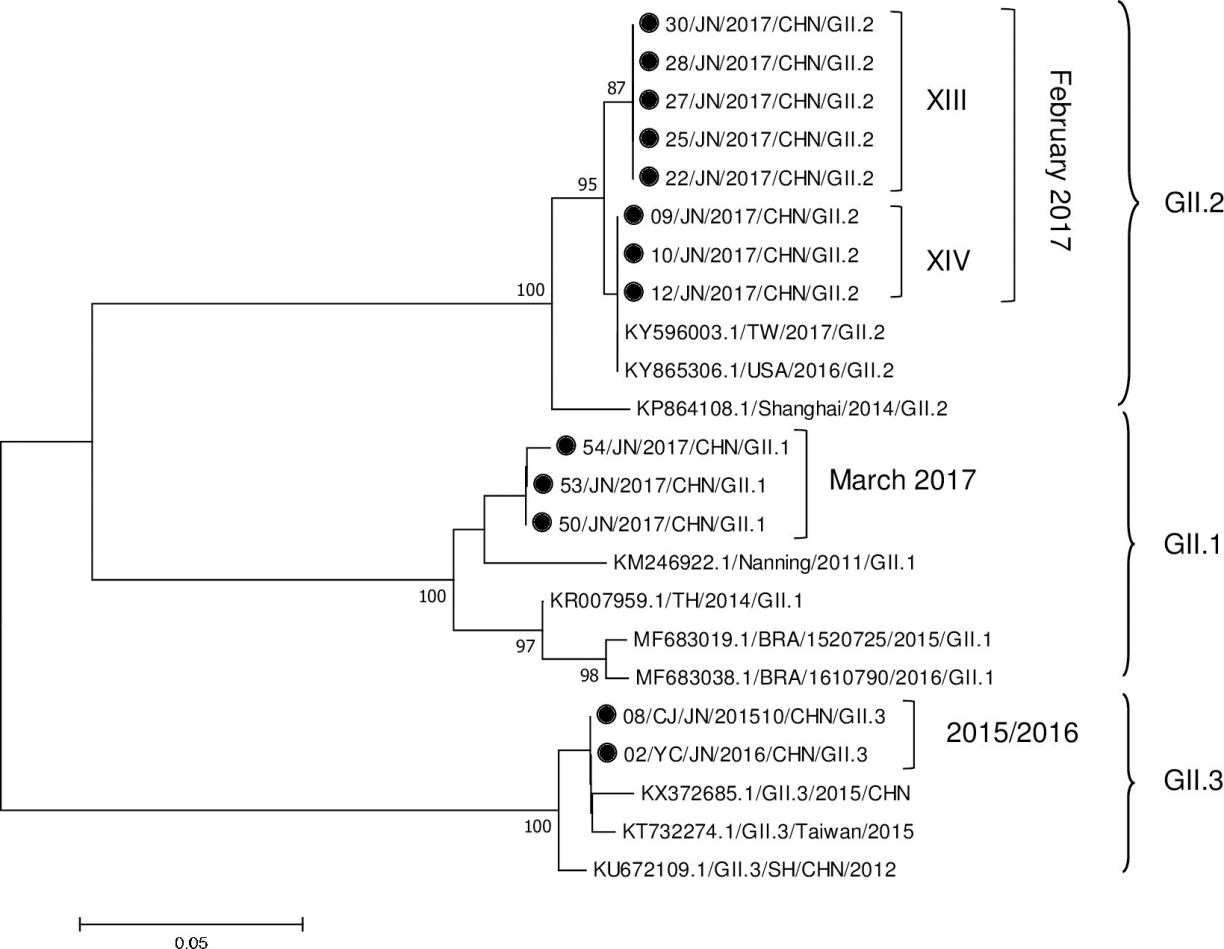
Phylogenetic analysis of GII.1, GII.2 and GII.3 norovirus strains isolated from outbreaks of acute gastroenteritis based on the partial sequence of the capsid gene encoding the VP1 protein. The phylogenetic tree was constructed using the neighbor-joining (N-J) method with the Kimura 2 parameter model and 1000 bootstrap replications. Bootstrap values are shown in the phylogenetic tree; values less than 80% are not represented. The bar at the bottom of the figure is proportional to the genetic distances. The black dot represents the NoV strains obtained from the outbreaks in Jinan in 2015–2017.

In addition, the other three GII genotypes were detected in the outbreaks. GII.1 and GII.2 were not detected in 2015–2016, but began to be found in the outbreaks occurring in the kindergartens in the first quarter of 2017 (Fig 4). GII.2 was found in the February outbreaks, and GII.1was identified in the March outbreak. The GII.2 strains from the outbreaks were grouped into two subclusters and originated from two infection sources (outbreaks XIII and XIV). All GII.2 strains showed high homology with the Taiwan strain from 2017 (KY596003), which suggested that the KY596003 strain might prevail in Taiwan and in Jinan, China. GII.1 had a higher homology with the Nanjing strain that appeared in 2011 (KM246922) than with the Thailand strain that appeared in 2014 (KR007959), indicating that the KM246922 strain appearing in 2011 re-emerged in 2017. GII.3 strains appeared in the outbreaks in October 2015 and March 2016. The GII.3 strains were more similar to the Anhui strain that appeared in 2015 (KX372685) than to the Pudong strain that appeared in 2012 (KU672109), which suggested temporal evolution of the GII.3 strains.

**Fig 5:**
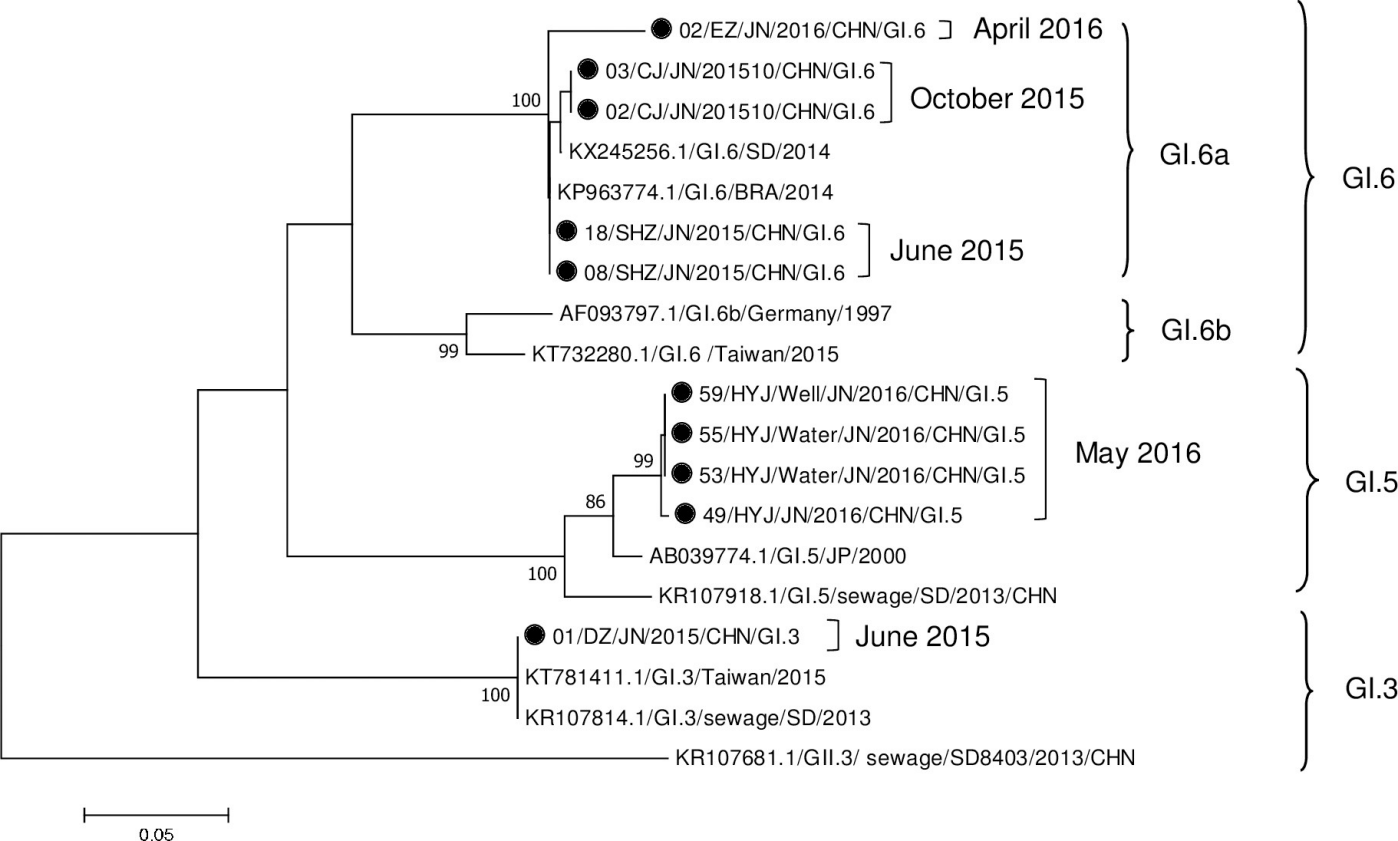
Phylogenetic analysis of GI norovirus strains isolated from outbreaks of acute gastroenteritis based on the partial sequence of the capsid gene encoding the VP1 protein. The phylogenetic tree was constructed using the neighbor-joining (N-J) method with the Kimura 2 parameter model and 1000 bootstrap replications. Bootstrap values are shown in the phylogenetic tree; values less than 80% are not represented. The bar at the bottom of the figure is proportional to the genetic distances. The black dot represents the NoV strains obtained from the outbreaks in Jinan in 2015–2016.

Three GI genotypes (GI.3, GI.5 and GI.6) were identified in the outbreaks. Phylogenetic analysis demonstrated that the GI.6 strains were grouped into 2 clusters (GI.6a and GI.6b) (Fig 5). The GI.6 strains from the outbreaks appeared to be from cluster GI.6a and had a close relationship with the Shandong strain from 2014 (KX245256), suggesting that the predominant GI.6 genotype appeared in China in 2014. All of the GI.5 strains, which were collected from water samples (well water and household tap water) and human specimens in outbreak X, showed high nucleotide sequence homology (99.7%-100%) with each other, implying that the well water was the most likely infection source in outbreak X.

## Discussion

Worldwide, NoVs are the leading cause of AGE outbreaks. Such outbreaks often occur in healthcare facilities, restaurants, cruise ships and institutional settings and have commonly drawn the attention of society. GII.4 has been successfully persistent, with periodic emergence over the past two decades worldwide (GII.4 US95/96 in 1995, GII.4 Farmington Hills in 2002, GII.4 Hunter in 2004, GII.4 Den Haag in 2006, GII.4 New Orleans in 2009, and GII.4 Sydney in 2012) [27]. However, our study focused on the occurrence of non-GII.4 NoVs and the important role of these NoVs in AGE outbreaks in Jinan, China.

The fifteen outbreaks reported to the Jinan CDC from June 2015 to March 2017 occurred mostly in colleges, kindergartens, and schools. Fourteen of the 15 outbreaks were caused by NoVs, including eight by GII infections and six by mixed infection with GII and GI/RoV-A. Seven predominant genotypes (GII.17, GII.3, GII.2, GII.1, GI.6, GI.5 and GI.3) were identified. Since late 2014, non-GII.4 genotypes have been sporadically associated with outbreaks. In the winter of 2014–2015, a novel GII.17 NoV strain (GII.17; Kawasaki 2014) emerged as a major causative agent of gastroenteritis outbreaks in China and Japan [17]. During November and December 2016, GII.2 was reported to be the most common genotype in Guangdong and Hong Kong, China [28–29]. During 2014 to 2015, GII.17, GII.3, GI.3, GI.5, and GI.6 were identified in NoV outbreaks in Victoria, Australia, but GII.4 was still the most common genotype detected [30]. In our study, no GII.4 strain was identified in the outbreaks. Thus, the emergence of diverse non-GII.4 genotypes played an important role in the outbreaks of AGE in Jinan, China.

The NoV VP1 protein is considered to be closely associated with the infectivity and antigenicity of NoV strains [31]. We conducted phylogenetic analysis on a partial VP1 region to understand the evolution of these non-GII.4 NoV strains. Two meaningful results emerged. First, the period of prevalence of each non-GII.4 NoV genotype varied. For example, GII.17 appeared and was the most common genotype in 2015, but its influence declined in 2016. In contrast, GII.2 and GII.1 did not appear until 2017. Second, the genetic characteristics of non-GII.4 NoVs continuously changed. The GI.6 strains identified in three outbreaks, all of which belong to subcluster GI.6a in the phylogenetic tree, had low nucleotide homology with the Taiwan strain from 2015, which belonged to subcluster GI.6b. Furthermore, the novel GII.17 variant was more similar to the Korea strain from 2016 than to the Korea strain from 2012.

The safety of drinking water has long been a worldwide health issue, especially in developing countries. Contaminated well water is an important transmission vehicle, and schoolchildren are susceptible to waterborne diseases in China [32]. A waterborne outbreak (outbreak X) in this study occurred in a rural residential district in Jinan. GI.5 strains isolated from the self-supply well water and the household tap water belonged to the same cluster as those isolated from infected residents. However, these findings regarding water contamination were different from those of previous reports. For example, GI.6 and GII.4 were found in Korean groundwater [33], and GII.17 accounted for 76% of the NoV contamination of surface water in Kenya [34]. Li and colleagues first reported an outbreak of GI.5 in Shanghai in February 2017 [35]. Outbreak X occurred in May 2016, prior to the outbreak in Shanghai. Thus, our study enriches the epidemiological data on NoV GI.5 outbreaks in China. Self-supply well water is very common in the rural district of Jinan, and the government should increase the supervision of self-supply well sanitation conditions to ensure access to safe water. In addition, waterborne viral outbreaks are often difficult to recognize. Sensitive methods are needed to detect viruses in environmental samples [36]. Our study showed that concentrating contaminated water via a centrifugal filter is an effective way to detect NoVs.

Ten outbreaks (outbreaks I-III, V-VIII and XIII-XV) in our study were associated with human-to-human transmission. These outbreaks occurred in colleges/kindergartens and had the common characteristic that the NoV-infected cases were not isolated in a timely manner. Li and colleagues reported that contact between students and individuals with suspected cases led to a 5.6-fold increase in the infection risk compared to the absence of contact with suspected cases [35]. Students and children are usually active and in close contact with others. Thus, the prompt identification of cases and the timely isolation of the NoV-infected cases is important to control NoV outbreaks.

The limitations of this study must be noted. First, although the AGE surveillance system has gradually improved in Jinan since 2015, epidemiological data on AGE outbreaks prior to 2015 were missing. Second, in order to control outbreaks as soon as possible, phylogenetic analyses were performed on the VP1 region of GI and GII NoVs. However, the rapid evolution of the VP1 gene has been speculated to result in immune-escape features and cause new pandemics [37]. Chan et al. noted that the emerging GII.17 NoV was identified by multiple genetic signatures of histo-blood group antigen (HBGA) binding and antigenic epitopes, had immune-escape features, caused severe gastroenteritis in previously less-vulnerable populations, and spread rapidly [38]. Furthermore, sequence analysis of the hypervariable P2 domain has been shown to be a powerful tool for genotyping and tracking outbreak-related samples [39–40]. Efforts to improve molecular analysis approaches for different NoV gene regions are underway.

## Conclusions

Our findings demonstrate that non-GII.4 NoV genotypes were the predominant circulating genotypes and were responsible for AGE outbreaks from 2015 to 2017 in Jinan, China. Non-GII.4 NoVs have been increasingly recognized as important causative agents of these outbreaks. In addition, the great diversity of the prevailing non-GII.4 strains, as well as the evolutionary features of these strains, demonstrated that the emergence of new NoV strains often leads to the occurrence of outbreaks. Thus, the implementation of year-round molecular epidemiological surveillance of NoVs is necessary to reduce the impact of NoVs on public health.
